# Effect of modified graded recession and anteriorization on unilateral superior oblique palsy: a retrospective study

**DOI:** 10.1186/s12886-017-0422-6

**Published:** 2017-03-14

**Authors:** Dong Cheol Lee, Se Youp Lee

**Affiliations:** Department of Ophthalmology, Keimyung University Dongsan Medical Center, Keimyung University school of Medicine, Daegu, 41931 South Korea

**Keywords:** Modified graded recession, Anteriorization, Inferior oblique muscle, Unilateral superior oblique palsy

## Abstract

**Background:**

Several inferior oblique (IO) weakening methods exist for correction of superior oblique palsy (SOP). A previously reported method involved recession and anteriorization according to IO overaction (IOOA) grade, which might be subjective and cause upgaze limitation and opposite vertical strabismus. Therefore, this study attempted to examine the efficacy of modified graded recession and anteriorization of the IO muscle in correction of unilateral SOP without resulting in upgaze limitation or opposite vertical strabismus.

**Methods:**

A total of 26 patients (male, 16; female, 10; age: 3–40 years) with SOP and head tilt or diplopia underwent modified graded recession and anteriorization. Patients were grouped by the position at which the IO muscle was attached inferior/temporal to the lateral border of the inferior rectus (IR) as follows: (1) 7.0/2.0 mm (4 patients), (2) 6.0/2.0 mm (3 patients), (3) 5.0/2.0 mm (3 patients), (4) 4.0/2.0 mm (11 patients), (5) 3.0/0.0 mm (2 patients), and (6) 2.0/0.0 mm (3 patients). Recession and anteriorization were matched to vertical deviation in the primary position at far distance. Remaining diplopia, head tilt, vertical deviation (≤3 prism diopter (PD), excellent; 4–7 PD, good; and ≥ 8 PD, poor), upgaze limitation, and opposite vertical strabismus were evaluated.

**Results:**

The average pre and postoperative 1-year vertical deviation angles in the primary position at far distance were 15.0 ± 5.6 PD and 1.2 ± 2.0 PD, respectively. At 1 year post-surgery, the vertical deviation angles were reduced by 6.8–21.0 PD from those at baseline. Few patients exhibited remaining head tilt, diplopia, upgaze limitation, or opposite vertical strabismus. Correction of hypertropia was excellent in 22 and good in 4 patients.

**Conclusions:**

Modified graded recession and anteriorization of the IO muscle is an effective surgical method for treating unilateral SOP. It exhibits good results and reduces the incidence of opposite vertical strabismus.

**Electronic supplementary material:**

The online version of this article (doi:10.1186/s12886-017-0422-6) contains supplementary material, which is available to authorized users.

## Background

Superior oblique palsy (SOP), the most common type of single-muscle paralytic strabismus, is congenital in about 40% of cases [1]. Acquired SOP is caused by diabetes, trauma, intracranial tumors, and vascular ischemia [2–5]. Clinical features include hypertropia in the paralyzed eye, increased vertical deviation upon adduction, and head tilt to the ipsilateral side. It is usually diagnosed in outpatient departments involved in maintenance of binocular vision. A long period of head tilting due to failure of binocular vision can lead to asymmetry of the face. There are many cases in which horizontal strabismus is accompanied by other manifestations apart from SOP. Therefore, SOP is diagnosed by a three-step test [6].

To determine the extent of surgery required, the degree of deviation in the primary or other positions and presence of inferior oblique muscle (IO) overaction (IOOA) and superior oblique muscle (SO) underaction should be considered. In case of IOOA, IO weakening surgeries, such as IO myotomy, myectomy, recession [7], anterior transposition [8], or disinsertion [9], are performed; in case of SO muscle underaction, SO reinforcement surgery, such as tucking in of the SO [10], has been proposed.

Guemes and Wright reported surgical anterior transposition of the IO muscle to the inferior rectus (IR) muscle insertion site. Graded anterior transposition involved reinsertion of the IO muscle at various points along the temporal aspect of the IR muscle, depending on the degree of IOOA [11].

However, the degree of IOOA may be a subjective parameter. Instead, objective determination of vertical deviation can be achieved by the alternate cover test in the primary position using a prism. Based on the method of Guemes and Wright [11], we investigated the effects of modified graded recession and anteriorization according to the degree of vertical deviation in the primary position at far distance as an objective method for evaluating the success of surgical outcomes without creating opposite vertical strabismus.

## Methods

The study design followed the tenets of the Declaration of Helsinki for biomedical research in human subjects. The institutional review board of the Keimyoung University Dongsan Medical Center approved this study. Since this was a retrospective study, informed consent was not required.

At our institution, from 2006 to 2015, surgical treatment for patients diagnosed with unilateral SOP with diplopia or head tilting involved modified graded recession and anteriorization in a step-by-step process. We performed a retrospective survey of medical records of 26 patients who had been followed up for more than 1 year [see Additional file 1].

Subjects with a history of ocular trauma or surgery, familial or acquired posterior segment diseases, congenital or progressive corneal diseases, neurological or systemic diseases, or history of strabismus surgery were excluded. Only subjects who had undergone accurate evaluation of deviation by the prism cover test by a single surgeon were included. This study included 16 male and 10 female patients diagnosed with SOP (age range, 3–40 years; mean, 10.92 ± 8.82 years; Table 1) [Additional file 2].

**Table 1:** Preoperative baseline characteristics of all participants

General characteristics	
Sex (M:F), n	16:10
Mean age, years	10.9 ± 8.8
IOOA grade	+1.9 ± 0.7
Preoperative average angle, PD	+15.0 ± 5.6

*IOOA* inferior oblique muscle overaction, *PD* prism diopter

Superior oblique palsy was diagnosed by the Parks three-step test [6], using the prism cover test and fundus examination when excyclotorsion was present. Vertical deviation was measured by the prism cover test at the primary position and at distances of one-third of a meter and 5 m. The up, down, right, and left deviations of gaze were measured at 5-m. The mean 5-m vertical deviation in the primary position, determined by a single surgeon as the average of three measurements for each patient, was used for analysis. Pre and postoperative severities of IOOA were graded by recording the differences in corneal limbus height between the two eyes of a patient, according to the degree of over-elevation of the eye upon adduction. Pre and postoperative over-elevation were scored on a scale ranging from 0 to +4.

**Fig. 1:**
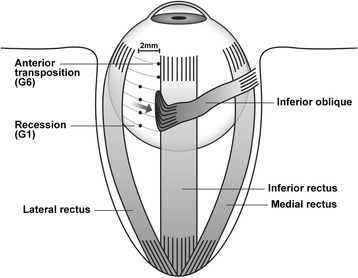
Surgical methods and points in each group. Surgery involved reinsertion of the IO muscle at various points along the temporal aspect of the IR muscle. Patients were categorized into six groups based on the inferior/temporal positions of attachment of the IO muscle with respect to the parallel axis of the IR lateral border. IO: inferior oblique; IR: inferior rectus; G6: group 6 (2.0/0.0 mm); G1: group 1 (7.0/2.0 mm)

**Fig. 2:**
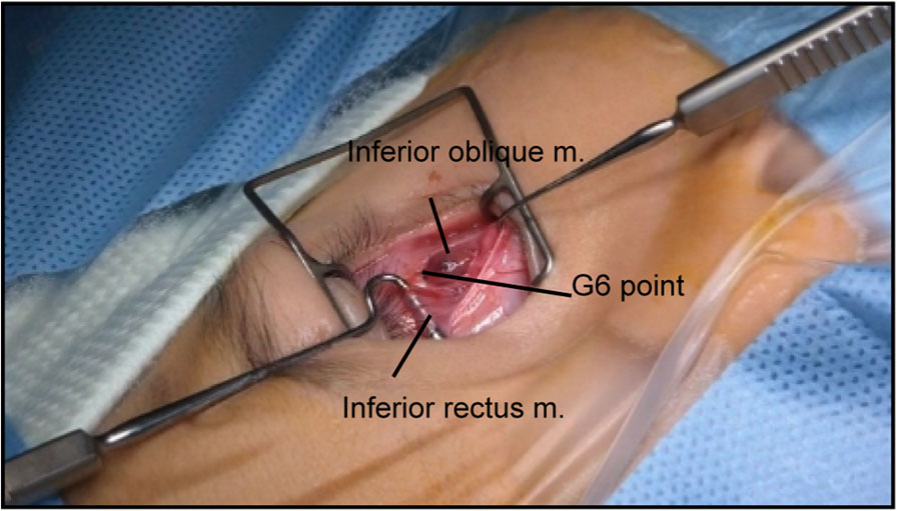
Surgery in group 6 (2.0/0.0 mm)

A single surgeon performed surgery for all patients, using a unique method involving a step-by-step surgery, whereby the IO muscle was weakened with anterior transposition of the IO muscle. In a novel approach for reducing upgaze limitation and opposite vertical strabismus, graded anterior transposition was performed according to the method of Guemes and Wright [11], based on the surgeon’s experience. This surgery involved reinsertion of the IO muscle at various points along the temporal aspect of the IR muscle. Patients were categorized into six groups based on the inferior/temporal positions of attachment of the IO muscle (anterior border and posterior border together as one point) with respect to the IR lateral border as follows: (1) 7.0/2.0 mm (*n* = 4), (2) 6.0/2.0 mm (*n* = 3), (3) 5.0/2.0 mm (*n* = 3), (4) 4.0/2.0 mm (*n* = 11), (5) 3.0/0.0 mm (*n* = 2), and (6) 2.0/0.0 mm (*n* = 3) (Table 2; Figs. 1 and 2).

**Table 2:** Surgical method and number of patients in each group

Surgical group (mm)^a^	Numbers of patients
7.0/2.0	4
6.0/2.0	3
5.0/2.0	3
4.0/2.0	11
3.0/0.0	2
2.0/0.0	3

^a^Surgery involved reinsertion of the IO muscle at various points along the temporal aspect of the IR muscle. Patients were categorized into six groups based on the inferior/temporal positions of attachment of the IO muscle (anterior border and posterior border together as one point) with respect to the IR lateral border. *IO* inferior oblique, *IR* inferior rectus, *G6* group 6 (2.0/0.0 mm), *G1* group 1 (7.0/2.0 mm)

The extent of surgery required was determined according to the required degree of postoperative vertical deviation in the primary position at far distance with IOOA as follows: (1) group 1, vertical deviation 6–8 prism diopter (PD); (2) group 2, vertical deviation 9–11PD; (3) group 3, vertical deviation 12–14 PD; (4) group 4, vertical deviation 15–17 PD; (5) group 5, vertical deviation 18–20 PD and (6) group 6, vertical deviation 21–23 PD. Surgical results were evaluated according to the presence or absence of diplopia or head tilt, remaining vertical deviation, and development of upgaze limitation and opposite vertical strabismus by the 1-year follow-up. Remaining vertical deviation in the primary eye position was graded as follows: ≤ 3 PD, excellent; 4–7 PD, good; and ≥ 8 PD, poor [12]. Normalization of head tilt or diplopia was graded based on the opinion of the outpatient department attendants.

Statistical analyses were performed using the Statistical Package for the Social Sciences version 18.0 software (SPSS, IBM Corp, Armonk, NY, USA). Pre and postoperative deviations were compared using the Mann–Whitney *U*-test.

A *P*-value < 0.05 was considered statistically significant.

## Results

A total of 26 patients were included in this study. The average preoperative angle of vertical deviation at far distance in the primary position and IOOA grade were 15.0 ± 5.6 PD and +1.9 ± 0.7 mm, respectively (*P* < 0.001). The postoperative follow-up period was 12 months. At the last follow-up, the average angle of vertical deviation at far distance in the primary position and IOOA grade were +1.2 ± 2.0 PD and +0.2 ± 0.3 mm, respectively (*P* < 0.001).

The average preoperative and 1-year postoperative angles of vertical deviation in each of the surgical groups are shown in Table 3. Only one patient in group 2 exhibited a remaining vertical deviation of 5 PD at the last follow-up. In group 4, 2 patients exhibited remaining vertical deviations of 5 and 6 PD at the last follow-up. One patient in group 5 exhibited remaining vertical deviation of 5 PD, while one in group 6 exhibited mild upgaze limitation at the last follow-up. The mean reductions in the angle of vertical deviation in the primary position in these groups were 6.8, 9.7, 12.7, 15.2, 17.0, and 21.0 PD, respectively.

**Table 3:** Mean reduction in angle of vertical deviation and IOOA grade post-surgery

Surgical group (mm)	Preoperative HT (PD)/IOOA	Postoperative HT (PD)/IOOA	Mean reduction HT (PD)/IOOA
7.0/2.0	+6.8/+0.9	0.0/0.0	6.8/0.9
6.0/2.0	+11.3/+1.0	+1.7/+0.2	9.6/0.8
5.0/2.0	+12.7/+2.3	0.0/0.0	12.7/2.3
4.0/2.0	+17.1/+2.1	+1.9/+0.3	15.2/1.8
3.0/0.0	+19.5/+2.3	+2.5/+0.5	17.0/1.8
2.0/0.0	+21.0/+2.7	0.0/0.0	21.0/2.7

*PD* prism diopter, *HT* hypertropia, *IOOA* inferior oblique muscle overaction

The mean preoperative and 1-year postoperative IOOA grades are shown in Table 3.

One year after surgery, none of the patients exhibited remaining head tilt or diplopia. Correction of hypertropia was excellent in 22 patients and good in 4 patients (groups 2, 4, and 5). Only one patient in group 6 exhibited upgaze limitation, and none of the patients exhibited vertical strabismus at the 1-year follow-up.

## Discussion

Knapp proposed the division of SOP into seven grades, with each grade requiring appropriate surgery. This has been considered as the basis for classification and treatment of the condition to date [13]. Surgical methods that weaken the IO muscle have been reported for treatment of SOP. Various IO muscle weakening surgeries have been reported since the first report of IO muscle tenotomy by Duane in 1906 [14]. In 1942, White first reported recession of the IO muscle [7]. Inferior oblique muscle weakening surgeries for IOOA include myotomy, disinsertion, denervation − extirpation, myectomy, anterior transposition, and various forms of recession [15, 16]. The choice of surgical method is determined in accordance with the experience and preference of the surgeon as well as the degree of IOOA. In general, although the above-mentioned surgeries are commonly used, in patients with an IOOA grade of +3, anterior transposition should be considered.

Elliott and Nankin reported successful anterior transposition of the IO muscle in patients with vertical deviation angles < 13 PD [8]. Engman et al. reported that anterior transposition of the IO muscle by suturing at 1, 2, and 3 mm anterior to the temporal border of the IR muscle can be used for the treatment of eyes with vertical deviation angles < 15 PD [17].

Patients with low IOOA are treated by recession rather than anterior transposition. However, recession is more challenging than myectomy and requires a longer operation time. Surgical myectomy is usually a short and simple procedure; however, the approach back to the ends of the broken muscle adhesions often results in recurrence of IOOA [18]. On the other hand, anterior transposition involves reinsertion of the IO muscle slightly forward along the temporal aspect of the IR muscle. It has been shown to be more effective than myectomy, particularly in patients with dissociated vertical deviation [19].

Guemes and Wright reported treatment of dissociative vertical deviation by concomitant recession of primary IOOA, unilateral SOP, and IOOA. This graded recession varied according to the severity of IOOA. Recession of the IO muscle was performed at 4 mm posterior and 2 mm temporal, 4 mm posterior, 3 mm posterior, 2 mm posterior, 1 mm posterior, or parallel to the temporal border of the IR muscle. Average corrections of 20, 18, and 15 PD were obtained, respectively, in patients who received 1, 2, and 3 mm posterior recessions [11].

In the present study, a single surgeon performed graded reattachment of the IO muscle to the posterior IR muscle according to the method of Guemes and Wright [11], but with some modifications. First, the extent of surgery performed in the present study was lower compared to that performed in the previous study. Moon and Lee, using the method of Guemes and Wright [11], reported IO muscle overaction in two eyes in which hypertropia developed in the opposite eye. They also reported the development of anti-elevation syndrome in patients who received 0 and 1-mm reattachments [20]. In the present study, none of the patients exhibited remaining head tilt or diplopia, although 4 patients in groups 2, 4, and 5 exhibited slight remaining hypertropia in the primary position, which was still categorized as a good outcome. At the 1-year follow-up, none of the patients exhibited upgaze limitation, except for one patient in group 6, or opposite vertical strabismus. Therefore, we deduced that the more conservative surgical approach used in the present study prevented upgaze limitation as well as hypertropia on the opposite site.

Secondly, Guemes and Wright used the extent of IOOA, which was determined subjectively, as a parameter to determine the extent of surgery [11]. In contrast, the present study employed the angle of vertical deviation in the primary position at far distance, which was determined objectively. This allowed the surgeon to determine the extent of surgery according to the angle of vertical deviation objectively, which allowed grouping.

## Conclusions

In summary, modified graded recession and anteriorization of the IO muscle might be suitable for treatment of patients with unilateral SOP with diplopia or head tilt and concomitant IOOA with angle deviation < 20 PD after objective examination of the vertical deviation in the primary position. This treatment method has minimal risk of upgaze limitation and hypertropia on the opposite side at 1 year post-surgery.

## Additional files

Additional file 1: SOP Dataset (2006–2015). SOP data of the 26 patients examined at our institution from 2006 to 2015. (XLSX 13 kb)
Additional file 2: Histogram of age distribution. SOP age distribution data of the 26 patients examined at our institution from 2006 to 2015. (DOCX 183 kb)

## Abbreviations

HT: Hypertropia
IO: Inferior oblique muscle
IOOA: Inferior oblique muscle overaction
IR: Inferior rectus
PD: Prism diopter
SO: Superior oblique muscle
SOP: Superior oblique palsy
