# Supplementary figures and images for: Effect of modified graded recession and anteriorization on unilateral superior oblique palsy: a retrospective study
Source: BMC Ophthalmol. 2017 Mar 14;17:27. doi: 10.1186/s12886-017-0422-6 (PMC5351157; doi:10.1186/s12886-017-0422-6)

Additional File 2

*Histogram of Age distribution

| Age (years) | N |
| --- | --- |
| 3 | 2 |
| 4 | 4 |
| 5 | 2 |
| 6 | 3 |
| 7 | 3 |
| 9 | 1 |
| 10 | 1 |
| 11 | 1 |
| 12 | 1 |
| 13 | 1 |
| 14 | 1 |
| 15 | 1 |
| 20 | 2 |
| 21 | 1 |
| 28 | 1 |
| 40 | 1 |


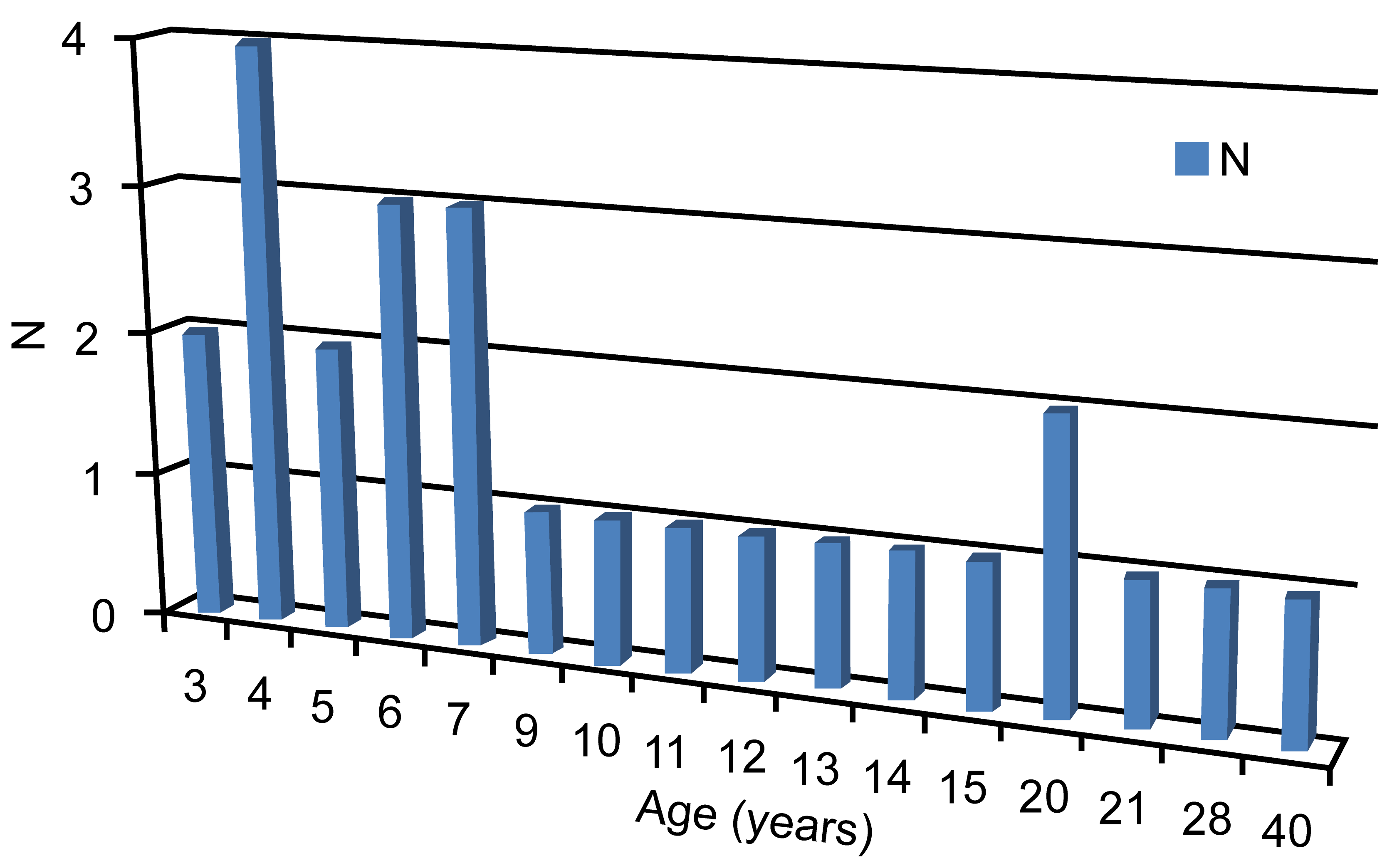

Supplement: Additional file 2: — Histogram of age distribution. SOP age distribution data of the 26 patients examined at our institution from 2006 to 2015. (DOCX 183 kb) [file 12886_2017_422_MOESM2_ESM.docx]
